# Multiple paternity assessed in the cuttlefish *Sepiella japonica* (Mollusca, Cephalopoda) using microsatellite markers

**DOI:** 10.3897/zookeys.880.33569

**Published:** 2019-10-14

**Authors:** Liqin Liu, Yao Zhang, Xiaoyu Hu, Zhenming Lü, Bingjian Liu, Li Hua Jiang, Li Gong

**Affiliations:** 1 National Engineering Laboratory of Marine Germplasm Resources Exploration and Utilization, College of Marine Sciences and Technology, Zhejiang Ocean University, Zhoushan 316022, China Zhejiang Ocean University Zhoushan China; 2 National Engineering Research Center for Facilitated Marine Aquaculture, Zhejiang Ocean University, Zhoushan 316022, China Zhejiang Ocean University Zhoushan China

**Keywords:** genetic diversity, mating, polyandry, reproductive strategy, sperm competition

## Abstract

Multiple paternity was demonstrated for seven clutches of eggs and 40 offspring sampled from these clutches in the cuttlefish *Sepiella japonica* from Fujian Shacheng Harbor Cultivation Base (Fujian Province, China), using four microsatellite DNA markers. It was observed that female cuttlefish copulated with different males. In this study, genotyping data suggest that at least three paternal allele genotypes were present in all seven clutches indicating that at least two males were responsible for each brood. Combined with behavioral observations, this study provides evidence for sperm competition and multiple paternity in *S. japonica*.

## Introduction

The cuttlefish *Sepiella japonica* Sasaki, 1929 (Mollusca, Cephalopoda) is a commercially important marine species in China. Production from wild stocks reached 60,000 tons in Zhejiang Province and accounted for more than 9.3% of provincial fishing catches in 1957 (Liu 2002; Wu et al. 2010). The resource of *S. japonica* has declined since the 1980s due to over-fishing and pollution (Jiang et al. 2014). To enhance production, artificial breeding methods are being developed in China and successful aquaculture techniques have been established in recent years (Yin et al. 2013). However, studies have revealed that the populations and individual genetic diversity in this species has declined under artificial conditions (Song and Wang 2009; Xu et al. 2011). The factors affecting the maintenance of genetic diversity have been a primary concern of conservation biologists.

An important factor that affects the genetic diversity of a population is the effective population size (Ne) which in turn is greatly influenced by the mating system of a species (Hoekert et al. 2002). The mating system influences Ne through changing the number of individuals contributing to subsequent generations (Brown et al. 2005). In a polyandrous mating system, females mate with several males within a single reproductive cycle in which the clustered offspring are descended from multiple males (Pearse and Anderson 2009). In such a mating system, Ne increases, and, as a result, maximizes the genetic diversity of the offspring within a single reproductive season (Sugg and Chesser 1994; Balloux and Lehmann 2003). Some studies have confirmed that a polyandrous mating system is frequent in marine cephalopods including *Octopus vulgaris* Sasaki, 1929 (Quinteiro et al. 2011), *Graneledone boreopacifica* Nesis, 1982 (Voight and Feldheim 2009), *Sepioteuthis australis* Quoy & Gaimard, 1832 (van Camp et al. 2004), *Sepia apama* Gray, 1849 (Naud et al. 2005), *Loligo pealeii* LeSueur, 1821 (Buresch et al. 2001), and *Loligo bleekeri* Keferstein, 1866 (Iwata et al. 2005). It is worth noting that the female of these species carries stored sperm from more than one male, and Ne will therefore be significantly higher (Pearse and Anderson 2009). Previous studies have shown that female *S. japonica* store sperm in the seminal receptacle found in the buccal membrane (Hanlon et al. 1999; Naud et al. 2005). All else being equal, long-term sperm storage enhances the opportunity for multiple matings of this species (Olsson et al. 1994; Ross 2001). Moreover, multiple matings of female *S. japonica* has actually been observed (Wada et al. 2006). Polyandry, coupled with sperm storage, is an important reproductive strategy for maximizing the genetic diversity of offspring in *S. japonica*.

In recent years, multiple paternity in several marine species has been documented using different genetic markers including allozymes, DNA fingerprinting, RAPDs, and microsatellites. Microsatellites are the preferred marker because they are widely distributed in the genomes of most organisms and are highly polymorphic (Jarne and Lagoda 1996). Paternity studies based on microsatellites have become increasingly common, and the number of studies using microsatellites has increased (Hoekert et al. 2002; Laloi et al. 2004; Takagi et al. 2008). Several microsatellite markers have been isolated and characterized for *S. japonica* and used to evaluate the genetic structure of its populations (Wu et al. 2010; Lü et al. 2017). In this study, we used the previously described microsatellite markers to investigate whether multiple paternity occurs in *S. japonica*. We observed multiple mating and paternity in this species and discussed the possible factors contributing to this reproductive strategy.

## Materials and methods

### Sample collection

Sexually mature adult *S. japonica* were obtained from the Fujian Shacheng Harbor Cultivation Base (Fujian Province, China). A sample of 200 wild adults was captured using traps and kept mixed into a cage (9 m^3^). Seawater parameters were continuously maintained at 25–27 °C and 23‰ salinity. From this sample, seven mating pairs were randomly chosen as breeders to produce the next generation. All behavioral interactions were recorded using closed-circuit television with infrared to observe individual animals. Each mating pair was gently captured and placed in a spawning tank until oviposition. Egg strings derived from each clutch were transferred to a hatchery tank. After hatching, 280 offspring were randomly collected for population genotyping, maintained in a tank until they reached a pre-determined age. The muscles from the mantle cavity of parents and offspring were taken and placed in 95% ethanol and stored at –20 °C until DNA extraction. Seven clutches (called A–G) were analyzed.

### DNA extraction and amplification

Total genomic DNA was isolated from each offspring and from the muscular tissue of the respective parents using the standard method of phenol-chloroform (Towner 1991). The concentration of DNA was estimated by a spectrophotometer (Nanodrop ND-2000, Thermo Electron Corporation, USA) and then the quality was assessed in 0.8% agarose gel. Three microsatellite loci, chosen from four loci (CL168, CL327, CL3354, CL904) developed specifically for *S. japonica* by Lü et al. (2017) were used to study genotypes for parents and their offspring.

The amplifications were carried out in a 2720 thermal cycler (ABI, USA) and in a 10 uL reaction volume: 2–10 ng DNA (0.5 µL), 0.5 µL of each forward and reverse primers, 5 µL 2×Es Taq MasterMix and 3.5 µL of double distilled water. The Polymerase Chain Reaction (PCR) conditions were initial denaturation for 5 min at 94 °C, followed by 30 cycles of denaturation for 40 s at 94 °C, annealing for 40 s at a primer-specific annealing temperature, extension for 40 s at 72 °C. PCR products were detected using capillary electrophoresis (BIOptic’s Qsep100 dna-CE, Taiwan) and allele size was estimated using Q-Analyzer software.

### Data analyses

Parents and their offspring were genotyped by determining alleles at three of the four microsatellite loci. We considered evidence from at least two loci to be necessary for estimation of multiple paternity, because evidence from one locus may have been caused by mutations or genotyping error (Davy et al. 2011). We determined paternal alleles through subtracting the maternal alleles from offspring in a brood following the technique of FitzSimmons (1998). The minimum number of sires for a clutch was assigned by counting the number of paternal alleles at each locus. Any instance of more than two possible paternal alleles at any loci indicated multiple paternity in a clutch (Buresch et al. 2001). In addition to manual reconstruction, we attempted to estimate paternal number, as genotypes, to corroborate our results using GERUD 2.0 (Jones 2005). Progeny genotypes were tested for conformity with Mendelian inheritance patterns using the X^2^ test (P < 0.05). Exclusion probabilities were assessed using the program CERVUS v. 2.0 (Marshall et al. 1998).

## Results

### Behavioral observations

**Figure 1.**
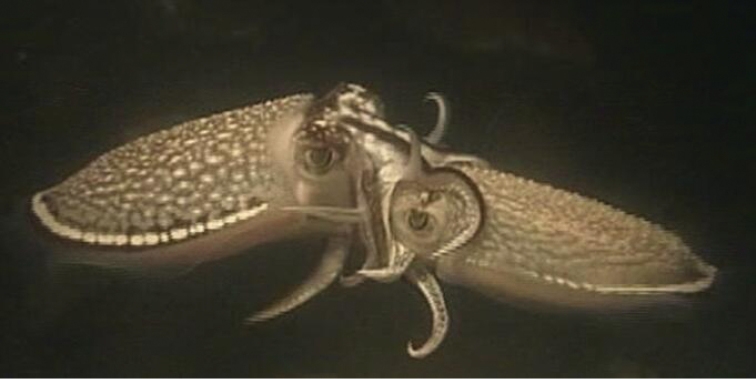
*S. japonica* mating in the head-to-head position.

Mating behavior in *S. japonica* involves courtship of a female by a male, and females may copulate with multiple males. Mating pairs mated in the head-to-head position during which males transfer spermatophores to the buccal membrane of the females or to an internal seminal receptacle (Fig. 1). The spermatophores that are deposited around the buccal area extrude the sperm mass to form spermatangium. Then the spermatangia attach to the buccal membrane where slowly released sperm are used for fertilization. We found that the male flushed water strongly when he was close to the female buccal area prior to mating with the female. This behavior is thought to dislodge sperm from previous males. We also found obvious courtship rituals and agonistic behaviors after sexual maturity. Males are generally capable of mating early in life (3–6 months maturity) and will continue to mate until senescence. However, the females do not generally lay eggs after copulating until fully mature. The duration of spawning in *S. japonica* varied from 21 to 30 days. Females lay multiple eggs (from tens to hundreds of thousands) by extruding them from the ovary and then they die shortly after spawning.

**Table 1.** Microsatellite loci used for paternity assessment in *Sepiella japonica*.

**Locus**	**Repeat motif**	**Primer Sequences(5’-3’)**	**Ta(°C)**	**GenBank Accession**
CL168	(AAC)_6_	F:ACAATCAACGGCTGTAAAGTCA	55	KU306816
		R:GACTATGGTTTGGATTTGGCAT		
CL3354	(CTG)_5_…(TGC)_5_	F:CCTCGGCTTCTGATGAAAAT	55	KU306828
		R:AGCCTTACTTCTGCAACATG		
CL904	(AT)_8_	F:TCTAGGCCTGTGGTTAATGT	55	KU306823
		R:TGATCGTTACTTGATGGCAG		
CL327	(TA)_6_	F: ACAGCATCTTCTGGTAAGCCAT	58	KX839255
		R: TAGTCCTGTCACCACAGTTATGC		

### Paternity analysis

Three of the four microsatellite markers were chosen to test paternity in seven offspring clutches. These loci exhibited three or more alleles and were polymorphic in each individual. We chose the locus which followed Mendelian inheritance to analyze paternity. Two hundred and eighty-seven individuals were genotyped at three loci, seven adult females and 280 offspring. The analysis was highly reproducible. We analyzed paternity including sampled males and non-sampled males that had copulated with females prior to capture. The exclusionary power of paternity assignments varied between 0.951 and 0.981. Maternal and offspring genotypes for each clutch are given in Table 2.

**Table 2.** Genotypes of maternal cuttlefish, offspring and estimated paternal cuttlefish of *Sepiella japonica*.

**Maternal Genotype**			**Offspring Genotype**					**Estimated Paternal Genotype**			
**Clutch Code**	**Locus**	**Genotype**	**I**	**II**	**III**	**IV**	**V**	**1**	**2**	**3**	**4**
A	CL168	155/170	155/160(21)	155/165(10)	175/170(9)			160/165	175/160		
	CL3354	240/260	240/250(3)	260/270(18)	240/230(19)			250/270	230/270		
	CL327	140/170	130/170(2)	140/160(15)	140/170(17)	160/170(6)		130/170	160/140		
B	CL168	175/185	175/180(12)	180/185(13)	185/200(5)	160/185(10)		180/180	180/200	160/200	
	CL3354	230/250	230/240(21)	235/250(9)	230/235(9)	230/250(1)	230/230(1)	240/230	235/235	240/235	
	CL327	160/160	145/160(16)	155/160(13)	150/160(11)			145/155	145/150	155/150	
C	CL168	150/160	140/150(14)	150/155(10)	140/160(12)	136/150(4)		140/140	140/136	150/136	
	CL3354	200/230	195/230(16)	195/200(6)	200/225(11)	210/230(3)	225/230(4)	200/195	230/195	195/225	
	CL327	140/154	136/140(13)	140/150(6)	136/154(13)	140/140(3)	150/154(5)	136/136	150/140	136/150	
D	CL168	160/180	175/180(13)	160/175(15)	165/180(5)	160/165(6)	160/160(1)	175/165	175/160	175/165	
	CL3354	220/240	220/235(14)	230/240(7)	255/240(2)	220/225(8)	220/245(9)	235/255	235/245	230/225	
	CL327	150/180	145/150(14)	160/180(6)	145/180(15)	150/160(1)	180/180(4)	145/160	145/160	160/180	
E	CL3354	260/270	250/260(29)	260/263(10)	260/265(1)			250/263	250/265		
	CL904	210/230	205/230(2)	200/210(4)	200/230(26)	205/210(4)	210/220(4)	200/220	205/200		
	CL327	140/160	135/140(24)	140/145(8)	135/160(4)	140/150(4)		135/135	145/150		
F	CL168	150/160	140/150(15)	140/160(8)	145/150(9)	150/180(7)	160/180(1)	140/180	145/145	140/145	
	CL3354	240/250	240/250(26)	240/240(1)	240/250(1)	250/260(10)	240/260(2)	250/260	240/250	240/260	
	CL904	220/230	220/230(9)	215/230(15)	210/230(9)	230/230(7)		215/210	230/215	230/210	
G	CL168	160/150	160/170(5)	140/160(20)	160/160(2)	150/160(4)	130/160(9)	170/160	170/150	150/130	130/140
	CL3354	220/250	240/250(16)	225/250(1)	220/240(12)	220/225(10)	220/250(1)	240/220	240/225	225/220	250/240
	CL904	260/280	250/280(7)	250/260(14)	260/270(15)	240/260(1)	260/280(2)	250/270	240/250	240/270	280/270

Notes: The numbers in the brackets represent number of offspring.

Almost all females were heterozygous at these loci (CL168, CL327, CL3354, CL904), except for CL327 (160/160) in the clutch B female. For clutches A and E, three different alleles which the father contributed were observed at the three chosen loci, suggesting that these two clutches had been sired by at least two males. The offspring of four females (B, C, D, and F) had three or four paternal alleles in each locus, and three paternal genotypes were observed in all loci. The number of paternal genotypes at these three loci indicated that females B, C, D, and F had mated with three different males. Within clutch G, five different alleles were detected at loci CL168 and CL3354, two of which were from maternal alleles. Clutch G showed four alleles for the locus CL904 in addition to the two alleles detected in the female. Four different paternal genotypes were estimated in clutch G, suggesting the female G was fertilized by at least four different males.

## Discussion

We observed female *S. japonica* mating with different males during the reproductive period, a behavior also recorded in other species of cephalopods (Hall and Hanlon 2002; Naud et al. 2005). The benefits of multiple mating not only may raise the potential for genetic diversity but also increases the possibility of offspring survival (Mann et al. 1966; Jennions and Petrie 2000). Female *Euprymna tasmanica* Pfeffer, 1884 that mated with different males had larger eggs than those that mated with one male, indicating that females may obtain nourishment from the seminal fluid of several males (Squires et al. 2012). Male cephalopods exhibit “flushing behavior” in which they remove fresh spermatangia from previous males (Hanlon et al. 1999). In *Sepia esculenta* Hoyle, 1885, the males remove sperm by using the hectocotylus instead of flushing water (Wada et al. 2005). The males in this study also exhibited such behavior, flushing the buccal area of the female with water, when mating with a previously mated female.

Microsatellite markers are particularly useful in paternity studies because of their polymorphism, codominance, and repeatability. Cephalopod biologists have determined multiple paternity in many species, including squid (van Camp et al. 2004; Shaw and Sauer 2004; Iwata et al. 2005), and *Graneledone boreopacifica* Nesis, 1982 (Voight and Feldheim 2009). In this study, at least three paternal allele genotypes were found in all seven clutches indicating that at least two males were responsible for each brood. This result was in accordance with that of Naud et al. (2004), where multiple paternity was also found in *Sepia apama*. Multiple paternity in *S. japonica* offspring indicates that sperm from different males must be mixed within the female’s reproductive tract. These sperm deposited around the buccal mass were used differentially to fertilize eggs (Shaw and Sauer 2004; Walker et al. 2006), after a process of sperm competition (Hanlon et al. 1999; Hall and Hanlon 2002) or mediation by female choice (Eberhard 1996).

Despite the prevalence of multiple paternity in cephalopod species, these studies show widely differing incidences of multiple paternity. In our study, multiple paternity was demonstrated in all sampled clutches (100%). In *Sepia apama*, one-third of the females mated with multiple males and 67% of females’ eggs had multiple sires (Naud et al. 2004). Several factors have been confirmed to be related to the variance in incidence of multiple paternity observed in cephalopod species, e.g., sperm allocation, mating systems, sperm competition, and female choice (Wada et al. 2005; Wada et al. 2010). Moreover, as suggested for the squid *Loligo bleekeri* by Iwata et al (2005), males who were the last to mate fertilized 85–100% eggs in four broods tested. However, in the multiple paternity study of *Loligo pealeii*, the mate order is not the most important factor in determining paternity (Naud et al. 2004; Buresch et al. 2009); however, no clear hypothesis has yet emerged to explain which factor is essential in the multiple paternity of *S. japonica*. Further work should be carried out to understand paternity patterns and to investigate different factors affecting multiple paternity in this species.

## References

[B1] Balloux F Lehmann L (2003) Random mating with a finite number of matings. Genetics 165: 2313–2315. 14704208 10.1093/genetics/165.4.2313 PMC1462883

[B2] Brown RC Woolliams JA McAndrew BJ (2005) Factors influencing effective population size in commercial populations of gilthead seabream, *Sparus aurata*. Aquaculture 247: 219–225. 10.1016/j.aquaculture.2005.02.002

[B3] Buresch KM Hanlon RT Maxwell MR Ring S (2001) Microsatellite DNA markers indicate a high frequency of multiple paternity within individual field-collected egg capsules of the squid *Loligo pealeii*. Marine Ecology Progress Series 210: 161–165. 10.3354/meps210161

[B4] Buresch KC Maxwell MR Cox MR Hanlon RT (2009) Temporal Dynamics of Mating and Paternity in the Squid *Loligo Pealeii*. Marine Ecology Progress Series 387: 197–203. 10.3354/meps08052

[B5] Davy CM Edwards T Lathrop A Bratton M Hagan M Henen B Nagy KA Stone J Scott Hillard L Murphy RW (2011) Polyandry and multiple paternities in the threatened Agassiz’s desert tortoise, *Gopherus agassizii*. Conservation Genetics 12: 1313–1322. 10.1007/s10592-011-0232-y

[B6] Eberhard WG (1996) Female Control: Sexual Selection by Cryptic Female Choice. Princeton University Press, New York, 501 pp.

[B7] Fitzsimmons NN (1998) Single paternity of clutches and sperm storage in the promiscuous green turtle (*Chelonia mydas*). Molecular Ecology 7: 575–584. 10.1046/j.1365-294x.1998.00355.x 9633101

[B8] Hall K Hanlon R (2002) Principal Features of the Mating System of a Large Spawning Aggregation of the Giant Australian Cuttlefish *Sepia Apama* (Mollusca: Cephalopoda). Marine Biology 140: 533–545. 10.1007/s00227-001-0718-0

[B9] Hanlon RT Ament SA Gabr H (1999) Behavioral aspects of sperm competition in cuttlefish, *Sepia officinalis* (Sepioidea: Cephalopoda). Marine Biology 134: 719–728. 10.1007/s002270050588

[B10] Hoekert WEJ Neufe´glise H Schouten AD Menken SBJ (2002) Multiple paternity and female-biased mutation at a microsatellite locus in the Olive Ridley sea turtle (*Lepidochelys olivacea*). Heredity 89: 107–113. 10.1038/sj.hdy.6800103 12136412

[B11] Iwata Y Munehara H Sakurail Y (2005) Dependence of Paternity rates on alternative reproductive behaviors in the squid *Loligo bleekeri*. Marine Ecology Progress Series 298: 219–228. 10.3354/meps298219

[B12] Jarne P Lagoda PJL (1996) Microsatellites, from molecules to populations and back. Trends. Ecology Evolution 11: 424–429. 10.1016/0169-5347(96)10049-5 21237902

[B13] Jennions MD Petrie M (2000) Why Do Females Mate Multiply? a Review of the Genetic Benefits. Biological Reviews 75: 21–64. 10.1017/S0006323199005423 10740892

[B14] Jiang LH Zhu AY Wu CW Su YQ Zhang JS Dong ZY (2014) Tetracycline Immersion tagging of cuttlefish, *Sepiella japonica*, larvae. Journal of the Word Aquaculture Society 45: 342–349. 10.1111/jwas.12116

[B15] Jones AG (2005) GERUD 2.0: a computer program for the reconstruction of parental genotypes from half-sib progeny arrays with known or unknown parents. Molecular Ecology Notes 5: 708–711. 10.1111/j.1471-8286.2005.01029.x

[B16] Laloi D Richard M Lecomte J Massotm M Clobert J (2004) Multiple paternity in clutches of common lizard *Lacerta vivipara*: data from microsatellite markers. Molecular Ecology 13: 719–723. 10.1046/j.1365-294X.2004.02102.x 14871374

[B17] Liu Y D (2002) General Situation of Fisheries Development in Cape Verde. Chinese Fisheries Economics 2: 1–50.

[B18] Lü Z M Hou L Gong L Liu LQ Chen YJ Guo BY Dong YH Wu CW (2017) Isolation and analysis on Est microsatellites of *Sepiella japonica* by novo high-throughput transcriptome sequencing. Oceanologia et Limnologia Sinica 48: 877–883.

[B19] Mann T Martin AW Thiersch JB (1966) Spermatophores and Spermatophoric Reaction in the Giant Octopus of the North Pacific, *OctopusDofleini Martini*. Nature 211: 1279–1282. 10.1038/2111279a0 5969810

[B20] Marshall TC Slate J Kruuk LEB Pemberton JM (1998) Statistical confidence for likelihood-based paternity inference in natural populations. Molecular Ecology 7: 639–655. 10.1046/j.1365-294x.1998.00374.x 9633105

[B21] Naud MJ Shaw PW Hanlon RT Havenhand JN (2005) Evidence for biased use of sperm sources in wild female giant cuttlefish (*Sepia apama*). Proceedings of the Royal Society B 72: 1047–1051. 10.1098/rspb.2004.3031 PMC1599881 16024363

[B22] Naud MJ Hanlon RT Hall KC Shaw PW Havenhand JN (2004) Behavioural and Genetic Assessment of Reproductive Success in a Spawning aggregation of the Australian Giant Cuttlefish, *Sepia apama*. Animal Behaviour 67: 1043–1050. 10.1016/j.anbehav.2003.10.005

[B23] Olsson M Gullberg A Tegelströ H (1994) Sperm competition in the sand lizard, *Lacerta agilis*. Animal Behaviour 48: 193–200. 10.1006/anbe.1994.1226

[B24] Pearse DE Anderson EC (2009) Multiple paternity increases effective population size. Molecular Ecology 18: 3124–3127. 10.1111/j.1365-294X.2009.04268.x 19555411

[B25] Quinteiro J Baibai T Oukhattar L Soukri A Seixas P Rey-Mendez M (2011) Multiple paternity in the common octopus *Octopus vulgaris* (Cuvier, 1797), as revealed by microsatellite DNA analysis. Molluscan Research 31: 15–20. 10.1007/s10750-017-3399-5

[B26] Ross KG (2001) Molecular ecology of social behavior: analyses of breeding systems and genetic structure. Molecular Ecology 10: 265–284. 10.1046/j.1365-294x.2001.01191.x 11298944

[B27] Shaw PW Sauer WHH (2004) Multiple Paternity and Complex Fertilisation Dynamics in the Squid *Loligo Vulgaris* Reynaudii. Marine Ecology Progress Series 270: 173–179. 10.3354/meps08052

[B28] Song WW Wang CL (2009) Genetic diversity of *Sepiella maindroni* in cultured and natural population. Oceanologia et Limnologia Sinica 40: 590–595.

[B29] Squires ZE Wong BBM Norman MD Stuart-Fox D (2012) Multiple Fitness Benefits of Polyandry in a Cephalopod. PLoS ONE 7: e37074. 10.1371/journal.pone.0037074 PMC3353885 22615896

[B30] Sugg DW Chesser RK (1994) Effective Population Sizes with Multiple Paternity. Genetics 137: 1147–1155. 7982568 10.1093/genetics/137.4.1147 PMC1206061

[B31] Takagi M Sakai K Taniguchi N (2008) Direct evidence of multiple paternities in natural population of viviparous Japanese surfperch by allelic markers of microsatellite DNA loci. Fisheries Science 74: 976–982. 10.1111/j.1444-2906.2008.01615.x

[B32] Towner P (1991) Purification of DNA. Essential molecular biology: a practical approach. In: Brown TA (Ed.) The Practical Approach Series, vol.1. Oxford University Press, Oxford, 47–68.

[B33] Voight JR Feldheim KA (2009) Microsatellite inheritance and multiple paternity in the deep-sea octopus *Graneledone boreopacifica* (Mollusca: Cephalopoda). Invertebrate Biology 128: 26–30. 10.1111/j.1744-7410.2008.00152.x

[B34] Van Camp LM Donnellan SC Dyer AR Fairweather PG (2004) Multiple Paternity in Field-and Captive-Laid Egg Strands of *Sepioteuthis Australis* (Cephalopoda: Loliginidae). Marine and Freshwater Research 55: 819–823. 10.1071/MF03179

[B35] Wada T Takegaki T Mori T Natsukari Y (2006) Reproductive behavior of the Japanese spineless cuttlefish *Sepiella japonica*. Venus 65: 221–228.

[B36] Wada T Takegaki T Mori T Natsukari Y (2010) Sperm Removal, Ejaculation and Their Behavioural Interaction in Male Cuttlefish in Response to Female Mating History. Animal Behaviour 79: 613–619. 10.1016/j.anbehav.2009.12.004

[B37] Wada T Takegaki T Mori T (2005) Sperm Displacement Behavior of the Cuttlefish *Sepia Esculenta* (Cephalopoda: Sepiidae). Journal of Ethology 23: 85–92. 10.1007/s10164-005-0146-6

[B38] Walker D Power AJ Sweeney-Reeves MJ Avise JC (2006) Multiple paternity and female sperm usage along egg-case strings of the knobbed whelk, *Busycon carica* (Mollusca; Melongenidae). Marine Biology 151: 53–61. 10.1007/s00227-006-0463-5

[B39] Wu CW Chi CF He GY Lü ZM Xu MY (2010) Isolation via enrichment and characterization of ten polymorphic microsatellite loci in the cuttlefish, *Sepiella maindroni* de Rochebruns. Acta Oceanologica Sinica 29: 121–124. 10.1007/s13131-010-0083-2

[B40] Xu MY Ye YY Guo BY Qi PZ Wu CW (2011) Optimization of the ISSR system for *Sepiella maindroni* and genetic diversity of the cultured population. Oceanologia et Limnologia Sinica 42: 538–542.

[B41] Yin F Sun P Peng SM Tang BJ Zhang D Wang CL Mu CK Shi ZH (2013) The respiration, excretion and biochemical response of the juvenile common Chinese cuttlefish, *Sepiella maindroni* at different temperatures. Aquaculture 402: 127–132. 10.1016/j.aquaculture.2013.03.018

